# Oxidative, epigenetic changes and fermentation processes in the intestine of rats fed high-fat diets supplemented with various chromium forms

**DOI:** 10.1038/s41598-022-13328-5

**Published:** 2022-06-14

**Authors:** Wojciech Dworzański, Ewelina Cholewińska, Bartosz Fotschki, Jerzy Juśkiewicz, Katarzyna Ognik

**Affiliations:** 1 Chair and Department of Human Anatomy, Medical University of Lublin, Jaczewskiego 4, 20-090 Lublin, Poland; 2 Department of Biochemistry and Toxicology, Faculty of Animal Sciences and Bioeconomy, University of Life Sciences in Lublin, Akademicka 13, 20-950 Lublin, Poland; 3 Institute of Animal Reproduction and Food Research, Polish Academy of Sciences, Tuwima 10, 10-748 Olsztyn, Poland

**Keywords:** Biochemistry, Immunology, Physiology, Biomarkers, Gastroenterology

## Abstract

The aim of the study was to determine how feeding rats a high-fat diet (F) supplemented with various forms of chromium affects the responses of the immune and redox systems, as well as epigenetic changes in the ileal tissue and the course of fermentation processes in the caecum. The rats received a pharmacologically relevant dose 0.3 mg Cr/kg body weight in form of chromium(III) picolinate (Cr-Pic), chromium (III)-methionine (Cr-Met), or chromium nanoparticles (Cr-NPs). The F increased DNA oxidation and raised the level of interleukin IL-6. The F was shown to reduce the intensity of fermentation processes in the caecum while increasing the activity of potentially harmful enzymes in the faeces. The addition of Cr in the form of Cr-NPs and Cr-Met in rats fed F beneficially increased mobilization of enzymes of the DNA repair pathway. All forms of Cr, but especially Cr-NPs, beneficially decreased the activity of caecal bacterial β-glucuronidase, faecal β-glucosidase and β-glucuronidase. However, due to the increase in level of cytokine IL-2 in small intestinal wall, induced by all tested forms of chromium, it is difficult to state conclusively that this element can mitigate unfavourable pro-inflammatory and oxidative changes induced by a F in the small intestinal wall.

## Introduction

Although for many decades Cr(III) was considered a key microelement for the proper functioning of the organism^1,2^, as a result of reassessment and revision of the available research results, the European Food Safety Authority (EFSA) concluded that this element is not necessary for proper functioning of the organism^3^. Also, Vincent^4^ in a later report confirms that Cr(III) is not an essential mineral and that it is not necessary to establish a dietary recommendation for the consumption of this element. According to Vincent^4^, Cr(III) administered to laboratory animals in supra-nutritional doses may have a beneficial effect on the organism, however, this has not been confirmed in clinical trials, in which usually proportionally smaller amounts of Cr(III) are used. Due to the fact that Cr(III) has the ability to regulate carbohydrate and lipid metabolism^5^, it is used in supplements intended for weight reduction^5,6^. There is no doubt that some of the consumers are taken mineral supplements carelessly. Supplements containing only chromium are freely available, and they commonly provide 200–500 µg chromium, although some contain up to 1000 µg^7^. This seems to be an easy way to consume high doses of Cr (III).

The results of the research carried out by Chen et al.^8^ showed that the positive effect of Cr on redox status can only be seen in glucose-intolerant individuals that regularly consume a high-fat diet, who are often obese. In healthy individuals with normal body mass, it can act as a pro-oxidant initiating free-radical reactions leading to oxidative stress^8^. This in turn can cause damage to important molecules, including proteins, lipids, and especially nucleic acids, and may also exacerbate inflammation in the body^9,10^. Moreover, the available literature indicates that although Cr(III) has not been shown to have genotoxic or mutagenic properties, it has the capacity to form coordination and covalent bonds with genetic material, which can lead to breaks in DNA strands and disrupt DNA stability, thereby directing cells onto a pathway of programmed cell death^1^. Thus, based on the often contradictory literature reports, it is difficult to state unequivocally whether the increasingly common addition of Cr(III) compounds to slimming preparations brings more benefits or health risks.

One of the important aspects that should be considered when analysing the potential benefits or risks of using Cr(III) as a dietary supplement is the form in which it occurs. Due to the fact that Cr in the form of inorganic compounds is relatively poorly absorbed from the gastrointestinal tract (only 0.9% in the case of chromium chloride), dietary supplements usually contain organic forms (mainly Cr picolinate), which are absorbed in the amount of 2.8%^5^. Attempts are being made to find alternative forms of Cr that would enable better utilization of this element and, as a consequence, it allowed to reduce its content in slimming preparations^9^. It is postulated that the use of a Cr complex with an amino acid such as methionine will increase its absorption in the gastrointestinal tract by changing the manner of its absorption in the small intestine from unsaturated passive transport via ion channels, characteristic of metal ions, to active transport via pathways used by amino acids^9,11^. Currently, however, scientists see the best chance at improving the bioavailability and utilization of Cr in increasingly popular Cr nanoparticles. They believe that due to their small size, lack of electric charge, and specific chemical and physical properties, these nanoparticles can better penetrate biological membranes and thus may be better absorbed from the gastrointestinal tract and utilized by the body^9–13^. On the other hand, the same properties can make nanoparticles potentially dangerous. There is a risk that chromium in the form of nanoparticles may increase oxidative processes in the body to a greater extent. It cannot be ruled out that chromium in the form of nanoparticles, undergoing oxidation processes in the body, is transformed into highly toxic Cr(VI).

In our previous research on rats fed a high-fat diet, supplementation with the pharmacologically relevant dose 0.3 mg/kg BW of Cr(III), particularly in the form of nanoparticles, increased DNA oxidation processes in the liver, heart, and brain, and also impaired DNA repair processes by reducing the activity of the endonuclease APE-1 in the blood^9,10^. Moreover, our previous research found that Cr nanoparticles and chromium picolinate have pro-apoptotic properties, as evidenced by increased levels of caspases 3 and 8 in the plasma of experimental rats. The use of Cr nanoparticles was also shown to impair the immune system by reducing the number of white blood cells^13^.

In light of the above, we postulated that since Cr(III), particularly Cr nanoparticles, negatively modulates the immune system and adversely affects the genetic material of the brain, heart, and liver, there is also a risk that these compounds can have harmful effects on the gastrointestinal tract. This may particularly affect the small intestine, which is exposed to contact with all substances from the external environment, and from which they are absorbed into the body. The aim of the study was to test this hypothesis by determining how feeding a high-fat diet to rats affects the responses of the immune and redox systems, as well as epigenetic changes in the ileal tissue and the course of fermentation processes in the caecum. An additional objective of the study was to establish whether and in what manner the addition of various forms of chromium at a pharmacologically relevant dose (0.3 mg/kg BW) to a high-fat diet affects these biological reactions in the intestines of laboratory rats.

## Results

### Effect of a high-fat diet in rats

During the eight-week experimental feeding, the rats treated with a high-fat, low-fibre diet had increased final body mass and decreased diet consumption in comparison to the animals fed the standard diet^10^.

In the rats receiving the high-fat diet, the villi of the small intestine were shorter than in the rats fed a standard diet (*P* = 0.049; Table 1). Levels of interleukin IL-2 and IL-6 in the small intestinal tissue were increased in rats fed the high-fat diet compared to the rats receiving a balanced diet (*P* = 0.047 and *P* = 0.027, respectively; Table 2). Administration of a high-fat diet to rats resulted in an increase in the content of 8-hydroxydeoxyguanosine (8-OHdG) and the activity of apurinic/apyrimidinic endonuclease (APE-1) in the tissue of the small intestine (*P* = 0.002 and *P* = 0.003, respectively; Table 3). Two-way ANOVA showed a significant decrease in the relative caecal tissue mass in rats receiving the high-fat, low-fibre diet (*P* < 0.001; Table 1). As shown in Table 4, irrespective of Cr supplementation, a significant increase in the extracellular activity of faecal bacterial β-glucuronidase followed the high-fat, low-fibre treatment on days 7, 28 and 56 (*P* < 0.05 vs. standard diet). In the caecal digesta, a similar increase was noted in the case of bacterial extracellular activity of β-galactosidase (Table 5; treatment F > treatment S; *P* < 0.05). Irrespective of Cr dietary supplementation, consumption of the high-fat, low-fibre diet caused a significant decrease in the caecal concentration of total SCFAs (*P* = 0.017), putrefactive SCFAs (*P* = 0.023), and acetic (*P* = 0.036), iso-butyric (*P* = 0.024), and valeric acids (*P* = 0.013) in comparison to the standard diet (Table 6).

**Table 1:** Final body mass, dietary intake, and small intestinal and caecal parameters of rats fed experimental diets.

	Body mass	Diet intake	Small intestine			Caecum		
	g	g/8 wks	Mass with digesta	Mean villus length	Mean crypt depth	Tissue mass	Digesta mass	Digesta pH
			g/100 g BM	μm	μm	g/100 g BM	g/100 g B	
**Group**								
S	375	988	1.54	429	140	0.151	0.381	7.61
F	417	902	1.53	387	137	0.136	0.328	7.71
SCr-Pic	363	973	1.57	419	144	0.163	0.261	7.65
FCr-Pic	428	908	1.51	401	141	0.138	0.267	7.66
SCr-Met	360	964	1.64	437	146	0.167	0.351	7.71
FCr-Met	428	925	1.52	413	140	0.134	0.306	7.46
SCr-NPs	362	980	1.51	377	129	0.147	0.327	7.66
FCr-NPs	413	890	1.51	374	131	0.130	0.321	7.71
SEM	4.888	7.148	0.014	0.234	0.089	0.003	0.013	0.035
**Cr source (Cr)**								
None	395	945	1.53	408	139	0.143	0.354	7.65
Cr-Pic	395	940	1.54	410	143	0.150	0.264	7.65
Cr-Met	394	944	1.58	425	142	0.150	0.328	7.58
Cr-NPs	387	935	1.51	375	130	0.138	0.324	7.68
*P value*	0.753	0.910	0.349	0.061	0.071	0.177	0.112	0.776
**Diet (D)**								
Standard (S)	365^b^	976^a^	1.56	415^a^	140	0.157^a^	0.330	7.66
High-fat (F)	422^a^	906^b^	1.52	394^b^	137	0.134^b^	0.305	7.63
*P value*	< 0.001	< 0.001	0.094	0.049	0.588	< 0.001	0.346	0.741
**Interaction (Cr × D)**								
*P value*	0.407	0.361	0.427	0.869	0.239	0.443	0.823	0.304

S—Rats were fed a standard diet; F—rats were fed a high-fat, low-fibre diet; SCr-Pic—rats were fed S diet supplemented with 0.3 mg Cr/kg BW from chromium(III) picolinate; FCr-Pic—rats were fed F diet supplemented with 0.3 mg Cr/kg BW from chromium(III) picolinate; SCr-Met—rats were fed S diet supplemented with 0.3 mg Cr/kg BW from chromium(III)-methionine; FCr-Met—rats were fed F diet supplemented with 0.3 mg Cr/kg BW from chromium(III)-methionine; SCr-NPs—rats were fed S diet supplemented with 0.3 mg Cr/kg BW from nano-sized chromium; FCr-NPs—rats were fed F diet supplemented with 0.3 mg Cr/kg BW from nano-sized chromium.

^a,b^Mean values within a column with unlike superscript letters were shown to be significantly different (*P* < 0.05); differences among the groups (S, F, SCr-Pic, FCr-Pic, SCr-Met, FCr-Met, SCr-NPs and FCr-NPs) were indicated with superscripts only in the case of a statistically significant interaction Cr × D (*p* < 0.05). SEM, pooled standard error of mean (standard deviation for all rats divided by the square root of rat number, n = 56).

**Table 2:** Immune status parameters of the ileal tissue of rats fed experimental diets.

	Globulins	IgA	IgG	IL-2	IL-6	TNF-α	Cp
	ng/g	ng/g	ng/g	pg/g	pg/g	pg/g	ng/g
**Group**							
S	398.78	39.67	45.62	1697.52	989.26	47.52	35.46
F	402.56	42.65	47.52	1739.45	1269.2	50.12	38.44
SCr-Pic	388.26	41.56	42.56	1725.63	1008.6	48.36	42.15
FCr-Pic	394.45	40.81	44.09	1899.62	1271.2	50.87	41.17
SCr-Met	412.32	37.64	44.36	1841.23	1124.5	49.92	38.56
FCr-Met	411.23	39.56	41.31	1917.14	1269.5	51.22	35.18
SCr-NPs	377.61	41.08	42.68	1896.2	1156.4	49.52	40.47
FCr-NPs	397.46	47.88	47.55	1899.2	1274.2	53.12	38.42
SEM	26.34	0.897	0.236	2.364	3.645	0.569	0.782
**Cr source (Cr)**							
None	400.67	41.16	46.57	1718.49^c^	1129.23	48.82	36.95
Cr-Pic	391.36	41.19	43.33	1812.63^b^	1139.90	49.62	41.66
Cr-Met	411.78	38.60	42.84	1879.19^a^	1197.00	50.57	36.87
Cr-NPs	387.54	44.48	45.12	1897.70^a^	1215.30	51.32	39.45
*P value*	0.369	0.772	0.127	0.047	0.366	0.298	0.059
**Diet (D)**							
Standard (S)	394.24	39.99	43.81	1790.15^b^	1069.69^b^	48.83	39.16
High-fat (F)	401.43	42.73	45.12	1863.85^a^	1271.03^a^	51.33	38.30
*P value*	0.782	0.069	0.174	0.047	0.027	0.339	0.845
**Interaction (Cr × D)**							
*P value*	0.136	0.288	0.817	0.512	0.288	0.947	0.647

S—Rats fed a standard diet; F—rats fed a high-fat, low-fibre diet; SCr-Pic—rats fed diet S supplemented with 0.3 mg Cr/kg BW from chromium(III) picolinate; FCr-Pic—rats fed diet F supplemented with 0.3 mg Cr/kg BW from chromium(III) picolinate; SCr-Met—rats fed diet S supplemented with 0.3 mg Cr/kg BW from chromium(III)-methionine; FCr-Met—rats fed diet F supplemented with 0.3 mg Cr/kg BW from chromium(III)-methionine; SCr-NPs—rats fed diet S supplemented with 0.3 mg Cr/kg BW from nano-sized chromium; FCr-NPs—rats fed diet F supplemented with 0.3 mg Cr/kg BW from nano-sized chromium.

^a,b,c^Means within a column with different superscript letters are significantly different (*P* < 0.05); differences between groups (S, F, SCr-Pic, FCr-Pic, SCr-Met, FCr-Met, SCr-NPs and FCr-NPs) are indicated with superscripts only in the case of a statistically significant interaction Cr × D (*p* < 0.05). SEM—pooled standard error of mean (standard deviation for all rats divided by the square root of the number of rats, n = 56).

IgA—Immunoglobulin A; IgG—immunoglobulin G; IL-2—interleukin 2; IL-6—interleukin 6; TNF-α—tumor necrosis factor alpha; Cp—ceruloplasmin.

**Table 3:** Oxidative and epigenetic DNA damage and activity of repair enzymes in ileal tissues of rats fed experimental diets.

	8-OHdG	Methylation	APE-1	TDG	ANPG
	pg/µL DNA	DNA, %	ng/g	ng/g	ng/g
**Group**					
S	39.45	11.56	1200.56	725.89	23.65
F	57.46	13.95	1436.98	689.97	24.15
SCr-Pic	41.25	11.84	1189.62	756.64	22.98
FCr-Pic	52.15	12.17	1357.26	745.21	20.87
SCr-Met	38.44	12.83	1223.2	825.14	23.41
FCr-Met	52.93	12.75	1209.3	798.62	21.48
SCr-NPs	43.64	15.23	1197.4	733.65	23.64
FCr-NPs	56.48	14.25	1539.2	705.24	20.89
SEM	0.647	0.127	5.369	1.468	0.367
**Cr source (Cr)**					
None	48.46	12.76	1318.77^b^	707.93^b^	23.90
Cr-Pic	46.70	12.01	1273.44^bc^	750.93^ab^	21.93
Cr-Met	45.69	12.79	1216.25^c^	811.88^a^	22.45
Cr-NPs	50.06	14.74	1368.30^a^	719.45^b^	22.27
*P value*	0.063	0.144	0.022	0.008	0.234
**Diet (D)**					
Standard (S)	40.70^b^	12.87	1202.70^b^	760.33	23.42
High-fat (F)	54.76^a^	13.28	1385.69^a^	734.76	21.85
*P value*	0.002	0.072	0.003	0.367	0.458
**Interaction (Cr × D)**					
*P value*	0.826	0.093	0.051	0.062	0.077

S—rats fed a standard diet; F—rats fed a high-fat, low-fibre diet; SCr-Pic—rats fed diet S supplemented with 0.3 mg Cr/kg BW from chromium(III) picolinate; FCr-Pic—rats fed diet F supplemented with 0.3 mg Cr/kg BW from chromium(III) picolinate; SCr-Met—rats fed diet S supplemented with 0.3 mg Cr/kg BW from chromium(III)-methionine; FCr-Met—rats fed diet F supplemented with 0.3 mg Cr/kg BW from chromium(III)-methionine; SCr-NPs—rats fed diet S supplemented with 0.3 mg Cr/kg BW from nano-sized chromium; FCr-NPs—rats fed diet F supplemented with 0.3 mg Cr/kg BW from nano-sized chromium.

^a,b,c^Means within a column with different superscript letters are significantly different (*P* < 0.05); differences between groups (S, F, SCr-Pic, FCr-Pic, SCr-Met, FCr-Met, SCr-NPs and FCr-NPs) are indicated with superscripts only in the case of a statistically significant interaction Cr × D (*p* < 0.05). SEM—pooled standard error of mean (standard deviation for all rats divided by the square root of the number of rats, n = 56).

8-OHdG—8-hydroxydeoxyguanosine; APE-1—apurinic/apyrimidinic endonuclease 1; TDG—thymine DNA glycosylase; ANPG—DNA-3-methyladenine glycosylase.

**Table 4:** Faecal extracellular bacterial β-glucosidase and β-glucuronidase activity throughout the feeding period in rats fed experimental diets^1^.

	Faecal β-glucosidase activity, µmol/h/g fresh faeces					Faecal β-glucuronidase activity, µmol/h/g fresh faeces				
	Day 0	Day 7	Day 14	Day 28	Day 56	Day 0	Day 7	Day 14	Day 28	Day 56
**Group**										
S	12.3	9.05^bc^	8.15^c^	7.41^bcd^	6.79^bc^	31.3	26.0	23.7^c^	25.3	23.9
F	12.0	13.2^a^	11.8^a^	12.4^a^	9.44^a^	31.7	37.7	43.2^a^	35.0	34.5
SCr-Pic	12.1	8.53^cd^	6.74^d^	6.68^cd^	5.70^c^	30.5	25.2	23.3^c^	21.2	21.9
FCr-Pic	12.1	9.79^bc^	8.99^bc^	8.41^bc^	7.53^bc^	30.5	33.3	32.2^b^	29.7	28.0
SCr-Met	12.2	9.19^bc^	6.91^cd^	6.58^cd^	6.28^c^	32.3	25.3	24.2^c^	22.5	21.2
FCr-Met	12.2	10.8^b^	10.5^ab^	8.97^b^	8.51^ab^	31.8	35.0	32.4^b^	32.0	30.7
SCr-NPs	12.1	6.86^de^	6.16^d^	6.18^d^	5.83^c^	30.7	22.1	19.2^c^	18.4	16.9
FCr-NPs	12.0	5.44^e^	3.89^e^	4.12^e^	3.55^d^	30.8	25.2	25.8^c^	26.4	24.1
*SEM*	0.334	0.377	0.399	0.385	0.309	0.960	0.961	1.188	1.072	1.098
**Cr source (Cr)**										
None	12.1	11.1	9.97	9.91	8.11	31.5	31.9^a^	33.4^a^	30.1^a^	29.2^a^
Cr-Pic	12.0	9.16	7.86	7.55	6.61	30.5	29.2^a^	27.7^b^	25.5^ab^	25.0^ab^
Cr-Met	12.1	10.0	8.73	7.78	7.40	32.0	30.1^a^	28.3^b^	27.3^ab^	26.0^a^
Cr-NPs	12.1	6.15	5.03	5.15	4.69	30.7	23.7^b^	22.5^c^	22.4^b^	20.5^b^
*P value*	0.999	< 0.001	< 0.001	< 0.001	< 0.001	0.946	< 0.001	< 0.001	0.021	0.012
**Diet (D)**										
Standard (S)	12.2	8.41	6.99	6.71	6.15	31.3	24.6^b^	22.6^b^	21.9^b^	21.0^b^
High-fat (F)	12.1	9.84	8.81	8.48	7.26	31.2	32.8^a^	33.4^a^	30.8^a^	29.3^a^
*P value*	0.941	0.005	< 0.001	< 0.001	0.016	0.991	< 0.001	< 0.001	< 0.001	< 0.001
**Interaction (Cr × D)**										
*P value*	0.997	0.002	< 0.001	< 0.001	< 0.001	0.998	0.160	0.016	0.983	0.810

S—rats fed a standard diet; F—rats fed a high-fat, low-fibre diet; SCr-Pic—rats fed diet S supplemented with 0.3 mg Cr/kg BW from chromium(III) picolinate; FCr-Pic—rats fed diet F supplemented with 0.3 mg Cr/kg BW from chromium(III) picolinate; SCr-Met—rats fed diet S supplemented with 0.3 mg Cr/kg BW from chromium(III)-methionine; FCr-Met—rats fed diet F supplemented with 0.3 mg Cr/kg BW from chromium(III)-methionine; SCr-NPs—rats fed diet S supplemented with 0.3 mg Cr/kg BW from nano-sized chromium; FCr-NPs—rats fed diet F supplemented with 0.3 mg Cr/kg BW from nano-sized chromium.

^a,b,c,d,e^Means within a column with different superscript letters are significantly different (*P* < 0.05); differences between groups (S, F, SCr-Pic, FCr-Pic, SCr-Met, FCr-Met, SCr-NPs and FCr-NPs) are indicated with superscripts only in the case of a statistically significant interaction Cr × D (*p* < 0.05). SEM—pooled standard error of mean (standard deviation for all rats divided by the square root of the number of rats, n = 56).

**Table 5:** Extracellular activity of bacterial enzymes in the caecal digesta of rats fed experimental diets, µmol/h/g digesta.

	α-glucosidase	β-glucosidase	α-galactosidase	β-galactosidase	β-glucuronidase
**Group**					
S	14.3	4.64^a^	9.95	48.3	21.3^b^
F	12.9	3.74^ab^	7.03	51.8	40.1^a^
SCr-Pic	14.8	3.76^ab^	8.38	40.8	25.3^b^
FCr-Pic	12.3	2.84^b^	7.76	58.7	25.6^b^
SCr-Met	13.0	2.43^b^	6.51	39.5	25.9^b^
FCr-Met	11.0	4.63^a^	7.83	55.5	20.2^b^
SCr-NPs	12.4	3.86^ab^	8.59	49.8	23.5^b^
FCr-NPs	9.33	2.76^b^	7.94	48.5	18.7^b^
SEM	0.646	0.188	0.321	2.022	1.236
**Cr source (Cr)**					
None	13.6	4.18	8.48	50.0	30.7
Cr-Pic	13.5	3.30	8.07	49.7	25.5
Cr-Met	12.0	3.52	7.17	47.5	23.0
Cr-NPs	10.9	3.31	8.21	49.1	21.0
*P value*	0.390	0.212	0.486	0.967	0.007
**Diet (D)**					
Standard (S)	13.6	3.67	8.36	44.6^b^	24.0
High-fat (F)	11.4	3.49	7.62	53.6^a^	26.1
*P value*	0.091	0.596	0.244	0.027	0.278
**Interaction (Cr × D)**					
*P value*	0.972	0.002	0.141	0.249	< 0.001

S—rats fed a standard diet; F—rats fed a high-fat, low-fibre diet; SCr-Pic—rats fed diet S supplemented with 0.3 mg Cr/kg BW from chromium(III) picolinate; FCr-Pic—rats fed diet F supplemented with 0.3 mg Cr/kg BW from chromium(III) picolinate; SCr-Met—rats fed diet S supplemented with 0.3 mg Cr/kg BW from chromium(III)-methionine; FCr-Met—rats fed diet F supplemented with 0.3 mg Cr/kg BW from chromium(III)-methionine; SCr-NPs—rats fed diet S supplemented with 0.3 mg Cr/kg BW from nano-sized chromium; FCr-NPs—rats fed diet F supplemented with 0.3 mg Cr/kg BW from nano-sized chromium.

^a,b^Means within a column with different superscript letters are significantly different (*P* < 0.05); differences between groups (S, F, SCr-Pic, FCr-Pic, SCr-Met, FCr-Met, SCr-NPs and FCr-NPs) are indicated with superscripts only in the case of a statistically significant interaction Cr × D (*p* < 0.05). SEM—pooled standard error of mean (standard deviation for all rats divided by the square root of the number of rats, n = 56).

**Table 6:** Concentration and profile of short-chain fatty acids in the caecal digesta of rats fed experimental diets.

	µmol/g								Profile, %		
	C2	C3	C4i	C4	C5i	C5	PSCFA	Total SCFA	C2	C3	C4
**Group**											
S	40.5	11.6	1.32	5.51^ab^	1.69	1.44	4.45	62.0	65.1	18.9	8.88^ab^
F	36.0	10.5	1.26	4.92^abc^	1.70	1.41	4.37	55.7	64.7	18.8	8.79^ab^
SCr-Pic	40.0	11.9	1.33	5.87^a^	1.86	1.50	4.70	62.5	64.0	19.1	9.29^a^
FCr-Pic	32.4	10.8	0.982	3.16^e^	1.36	1.14	3.49	49.9	64.8	21.9	6.22^c^
SCr-Met	37.6	11.5	1.21	4.21^bcde^	1.63	1.47	4.30	57.6	65.2	20.0	7.27^bc^
FCr-Met	34.3	10.8	1.01	3.97^cde^	1.40	1.16	3.57	52.7	65.3	20.5	7.49^bc^
SCr-NPs	35.0	10.5	1.24	4.71^abcd^	1.64	1.36	4.24	54.5	64.2	19.3	8.62^ab^
FCr-NPs	35.0	10.7	1.11	3.33^de^	1.58	1.19	3.88	52.9	66.1	20.3	6.23^c^
SEM	0.902	0.234	0.041	0.196	0.054	0.043	0.131	1.321	0.343	0.291	0.231
**Cr source (Cr)**											
None	38.2	11.0	1.29	5.21	1.69	1.42	4.41	58.8	64.8	18.8	8.83
Cr-Pic	36.2	11.3	1.15	4.51	1.60	1.32	4.09	56.1	64.3	20.5	7.75
Cr-Met	35.9	11.1	1.10	4.09	1.51	1.31	3.93	55.1	65.2	20.2	7.37
Cr-NPs	35.0	10.6	1.17	4.01	1.61	1.27	4.06	53.7	65.1	19.8	7.42
*P value*	0.637	0.751	0.425	0.049	0.683	0.634	0.600	0.543	0.818	0.145	0.026
**Diet (D)**											
Standard (S)	38.3^a^	11.3	1.28^a^	5.07	1.70	1.44^a^	4.42^a^	59.1^a^	64.6	19.3	8.51
High-fat (F)	34.4^b^	10.7	1.09^b^	3.84	1.50	1.22^b^	3.82^b^	52.8^b^	65.2	20.4	7.18
*P value*	0.036	0.183	0.024	< 0.001	0.075	0.013	0.023	0.017	0.411	0.057	< 0.001
**Interaction (Cr × D)**											
*P value*	0.514	0.754	0.601	0.048	0.366	0.519	0.446	0.496	0.698	0.284	0.005

S—Rats fed a standard diet; F—rats fed a high-fat, low-fibre diet; SCr-Pic—rats fed diet S supplemented with 0.3 mg Cr/kg BW from chromium(III) picolinate; FCr-Pic—rats fed diet F supplemented with 0.3 mg Cr/kg BW from chromium(III) picolinate; SCr-Met—rats fed diet S supplemented with 0.3 mg Cr/kg BW from chromium(III)-methionine; FCr-Met—rats fed diet F supplemented with 0.3 mg Cr/kg BW from chromium(III)-methionine; SCr-NPs—rats fed diet S supplemented with 0.3 mg Cr/kg BW from nano-sized chromium; FCr-NPs—rats fed diet F supplemented with 0.3 mg Cr/kg BW from nano-sized chromium.

^a,b,c,d,e^Means within a column with different superscript letters are significantly different (*P* < 0.05); differences between groups (S, F, SCr-Pic, FCr-Pic, SCr-Met, FCr-Met, SCr-NPs and FCr-NPs) are indicated with superscripts only in the case of a statistically significant interaction Cr × D (*p* < 0.05). SEM—pooled standard error of mean (standard deviation for all rats divided by the square root of the number of rats, n = 56).

SCFA—Short-chain fatty acids; PSCFA—putrefactive SCFA (the sum of iso-butyric, iso-valeric and valeric acids); acids: C2—acetic; C3—propionic; C4i—iso-butyric; C4—butyric; C5i—iso-valeric; C5—valeric.

### Effect of different dietary forms of Cr in rats

Among the rats receiving a chromium supplement in their diet during the eight-week experimental period, final body mass was lower only in the group receiving Cr-NPs, in comparison with the group whose diet did not contain a chromium supplement^10^.

The addition of chromium to the diet, irrespective of the form used, increased the IL-2 (*P* = 0.047) level in the small intestinal tissue relative to rats fed diets without additional Cr. This effect was most pronounced in the groups receiving Cr-Met and Cr-NPs (Table 2). Irrespective of diet type, the addition of Cr-Met to the diet of rats decreased the level of APE-1 (*P* = 0.022) in the ileal tissue, whereas the addition of Cr-NPs increased its level relative to the rats fed a diet without added Cr. In addition, an increase in the content of thymine DNA glycosylase (TDG; *P* = 0.008) in the ileal tissue was observed in the rats whose diet was supplemented with Cr-Met in comparison to the treatments without Cr and with Cr-NPs (Table 3). Irrespective of the diet type, dietary supplementation with Cr-NPs caused a significant decrease in extracellular bacterial β-glucuronidase activity in the faeces collected on day 7 (*P* < 0.05 vs. other treatments), day 28 (*P* < 0.05 vs. treatment without additional Cr), and day 56 (*P* < 0.05 vs. treatments without Cr and with Cr-Met) (Table 4).

### Interaction between diet type and Cr form

As shown in Table 4, a significant Cr × D interaction caused an increase in the extracellular activity of bacterial faecal β-glucosidase on day 7 in the rats from group F versus the control group S (*P* < 0.05). This effect was not observed between the counterparts fed the same form of dietary Cr, i.e. SCr-Pic versus FCr-Pic; SCr-Met versus FCr-Met; and SCr-NPs versus FCr-NPs (in all cases *P* > 0.05). The Cr × D interaction was also such that the lowest β-glucosidase activity on day 7 was noted in the FCr-NPs rats, differing significantly from all groups except SCr-NPs. A Cr × D interaction was additionally observed for faecal β-glucosidase activity on days 14, 28 and 56. Interestingly, comparison of counterparts fed the same dietary Cr form or a diet without added Cr (groups S and F) showed higher activity of that bacterial enzyme in the high-fat, low-fibre groups, except those treated with Cr-NPs. As a result, the lowest activity of β-glucosidase was noted in the faeces of rats fed a high-fat, low-fibre diet supplemented with Cr-NPs (*P* < 0.05 vs. all remaining groups). A Cr × D interaction was also noted for faecal β-glucuronidase activity on day 14, with the highest activity following consumption of the F diet (*P* < 0.05 vs. all other groups). Additionally, for both control groups and for Cr-Pic and Cr-Met, but not for the Cr-NPs groups, higher activity of bacterial faecal β-glucuronidase was obtained following the high-fat, low-fibre treatments (*P* < 0.05; F > S; FCr-Pic > SCr-Pic; FCr-Met > SCr-Met). A Cr × D interaction was noted for extracellular activity of bacterial β-glucosidase and β-glucuronidase in the caecal digesta (Table 5). Comparison of groups fed the same form of dietary Cr showed that caecal β-glucosidase activity was significantly higher in the FCr-Met rats than in the SCr-Met group, while this effect was not observed in the controls (S vs. F) or in the Cr-Pic and Cr-NPs groups. In the case of extracellular activity of β-glucuronidase, the Cr × D interaction was such that this activity was highest in the caecal digesta of the F rats (*P* < 0.05 vs. all other groups). The lowest caecal concentration of butyric acid was observed in the FCr-Pic group, and it differed significantly from its dietary counterpart SCr-Pic (*P* < 0.05). No such effect was noted for the other dietary counterparts (S vs. F; SCr-Met vs. FCr-Met; SCr-NPs vs. FCr-NPs; Table 6; see significant Cr × D interaction, *P* = 0.048). In the case of the butyric acid profile in the caecal digesta, the Cr × D interaction revealed that groups FCr-Pic and FCr-NPs, but not groups F and FCr-Met, differed significantly from their counterparts fed a standard diet. Additionally, in the dietary high-fat, low-fibre treatments, Cr-Pic and Cr-NPs lowered the butyric acid concentration and profile in the caecal digesta in comparison to the control F group, but Cr-Met did not.

## Discussion

The available literature indicates that the use of a high-fat diet results in the development of chronic systemic low-grade inflammation, as well as localized tissue dysfunction^14,15^. Inflammation mainly affects the nervous system, liver, adipose tissue, skeletal muscles, and above all the intestines^16^. This is because the gastrointestinal tract is the organ most exposed to various components of the diet, including high fat content, toxins, or food antigens^15^. Previous observations suggest that induction of inflammation by high fat intake in the diet is directly linked to the development of a number of intestinal diseases, including inflammatory bowel disease (Crohn’s disease or ulcerative colitis)^14–16^. It is believed that a high-fat diet, by directly stimulating pro-inflammatory signalling cascades and indirectly increasing levels of barrier-disrupting cytokines such as tumour necrosis factor α (TNFα), interleukins IL1B and IL6, and interferon γ (IFNγ), damages the intestinal barrier via changes in the conformation of tight junction proteins regulating transport of digesta from the intestinal lumen to the bloodstream, thereby increasing intestinal permeability^17,18^. IL-6 has been shown to modulate the intestinal immune response by increasing expression of adhesion factors of endothelial cells, thereby enabling migration of phagocytes and lymphocytes to sites of inflammation^19^. IL-2 is responsible for activating and controlling the immune response to inflammation by stimulating the activation and proliferation of T cells (especially Tc and Treg subpopulations) and NK cells, stimulating B cells to produce antibodies, and promoting apoptotic cell death, mediated by Fas ligand^20^. The results of the present research on rats confirm the pro-inflammatory effect of a high-fat diet on the small intestine, as indicated by the increased levels of both IL-2 and IL-6. A high-fat diet is thought to stimulate the release of cytokines by directly acting on the enterocytes of the small intestine as well as by activating their production by macrophages and mast cells of the lamina propria^21^. In addition, inflammatory cytokines produced in adipose tissue accumulating in the body due to long-term consumption of a high-fat diet can reach the epithelial layer of the intestines^22^. Moreover, previous research of Dworzański et al.^13^ showed an increase in plasma levels of IL-6 and TNF-α in rats administered a high-fat diet for eight weeks. This suggests that the inflammation induced by a high-fat diet is not limited to the small intestine, but is systemic.

It should be noted that a high-fat diet may induce inflammation not only directly by increasing synthesis of pro-inflammatory cytokines, but also indirectly through increased synthesis of free oxygen radicals. Free fatty acids undergo mitochondrial beta-oxidation. The higher their quantity in the diet, the greater is the flow of electrons through cytochrome c, resulting in excessive production of free oxygen radicals. These in turn readily react with subsequent lipids, leading to their peroxidation^23^. The results of a previous research by Dworzański et al.^9^, in which rats received a high-fat diet for eight weeks, showed increased content of malondialdehyde in the plasma, which seems to fully confirm this assumption^9^. Furthermore, it is believed that increased free radical synthesis during the use of a high-fat diet may be linked to increased production and secretion of bile acids needed for fat emulsification. Araki et al.^24^ showed that treating Caco-2 cells with hydrophobic fatty acids at higher than physiological concentrations resulted in an increase in synthesis of reactive oxygen species (ROS) due to stimulation of phosphoinositide 3-kinase (PI3K). Increased ROS levels in the body induce overexpression of pro-inflammatory cytokines, including nitrogen oxide (NO), tumour necrosis factor alpha (TNF-α), and interferon gamma (IFN-γ), leading to the development of inflammation^23^.

Overproduction of free oxygen radicals induced by a high-fat diet, however, may not only induce inflammation but also lead to extensive damage to cellular macromolecules, including DNA^9^. This results in DNA strand breaks, and above all to modifications of nitrogen bases, especially guanine^25^. The previous research of Dworzański et al.^9^ showed increased oxidation of genetic material in the heart and brain of rats receiving a high-fat diet for eight weeks^9^. Damage to genetic material by free radicals can lead to serious mutations disturbing genome integrity, and even initiating tumour development^25^. To prevent this, or at least significantly reduce the risk, the body has developed multiple DNA repair mechanisms. When abnormal modification of guanine or another nitrogen base takes place, the base excision repair (BER) pathway is activated^26^. This mechanism enables the excision of a single damaged base and its replacement with a new one with normal structure. This process takes place in several stages. First, the appropriate DNA glucosidase cleaves the β-N-glycosidic bond, and then removes the damaged base, creating an AP site (apurinic or apyrimidinic). This site is recognized by endonuclease APE-1, which cleaves the phosphodiester bond on the 5’ side of the lesion, creating free 3’-OH terminals. DNA polymerase binds to these terminals and fills in the gap with a new strand synthesized on the DNA template. In the final stage of DNA repair, the two strands are joined by a phosphodiester bond, whose formation is catalysed by DNA ligase^27^. The present study showed an increased level of 8-OHdG in the wall of the small intestine of rats fed a high-fat diet, accompanied by an increase in APE-1 activity. It can therefore be concluded that the high-fat diet induced oxidative stress leading to guanine damage in the cells of the small intestine, which in turn activated repair pathways, as evidenced by the increase in APE-1 activity.

The negative effect of a high-fat diet on the small intestine of rats in the present study seems to be confirmed by the results of the histological examination, as the length of the villi was decreased in rats fed a diet rich in fat. Similar results were reported by Xie et al.^28^, who administered a high-fat diet to 12-month-old mice for 14 weeks. The results are difficult to interpret. However, Yamamoto et al.^29^, in a study on mice, demonstrated that during application of a high-fat diet, the ability to absorb fat in the gastrointestinal tract declined with age, which was accompanied by a decrease in villus length. Thus it is possible that this process may explain the decrease in villus length noted in the present study and by Xie et al.^28^. It is also possible that the decrease in villus length caused by a high-fat diet is a type of defence mechanism developed by the body in order to limit absorption of fat and thus its harmful effects on the body.

There is emerging evidence that a high-fat diet causes considerable changes in the microbial composition of the large intestine, decreasing its diversity, as well as changes in enzymatic and metabolic activity^30^. Modulations of large intestinal microbial communities induced by a high-fat diet are often accompanied by metabolic disturbances, such as intestinal permeability and inflammation or increased lipogenesis and adipogenesis^31^. Many authors have reported a decrease in *Bacteroidetes* and an increase in *Firmicutes* and *Proteobacteria* following intake of a high-fat diet in laboratory rodents^30^. In presented study, a high-fat, low-fibre diet was used to induce anticipated adverse effects at the small and large intestinal level. This type of diet had been used successfully in our previous experiments on rats^32,33^. For analysis of intestinal microbial activity, the present study focused on the caecum, as the main site of bacterial fermentation in rats. We decided to analyse the concentration and profile of the major end-products of bacterial fermentation (short-chain fatty acids) as well as the extracellular activity of selected bacterial enzymes in the faeces (during the feeding period) and caecal digesta. In the faeces collected on days 0, 7, 14, 28 and 56, we focused on two potentially harmful bacterial enzymes, β-glucosidase and β-glucuronidase. The group of caecal enzymes involved in conversions of endogenous toxins and genotoxic compounds comprises azoreductase, nitroreductase, β-glucuronidase, β-glucosidase, β-galactosidase, and 7-α-dehydroxylase. *Bacteroides* sp., *Enterococcus* sp., *Clostridium* sp., and *Eubacterium* sp. are efficient producers of some of the aforementioned glucosidases (β-glucosidase, β-glucuronidase, and β-galactosidase) with potentially harmful effects on the host^34^. B-glucosidase and β-glucuronidase have been reported to be present in *Bacteroidetes* and *Firmicutes*^34–36^, but especially high activity of β-glucuronidase followed increased growth of harmful *Escherichia coli* and *Enterobacteriaceae* species^37^. In the present study, the high-fat, low-fibre diet resulted in a significant increase in the extracellular activity of bacterial β-glucuronidase and β-glucosidase in the faeces of rats during the entire feeding period (measurements on days 7, 14, 28 56), as well as an increase in β-glucuronidase and β-galactosidase activity in the caecal digesta measured at the termination of the study (day 56 of feeding). These results confirmed that an overflow of dietary lard to the caecum, and then to the colon and forming faeces, leads to adverse changes in the enzymatic activity of large bowel microbes. An additional effect observed upon dietary application of a high-fat, low-fibre diet was diminished concentration of short-chain fatty acids produced by the microbiota in the caecum of rats. This in turn may have contributed to the reduction observed in relative caecal tissue mass, despite the unchanged relative digesta mass in that segment of the large gut. The amount and types of SCFAs are a biomarker of the health status of the gut and of the entire body^38^. An increase in minor, putrefactive SCFAs should be regarded as an indication of adverse fermentation processes in the lower GIT in a specific dietary environment. One of the beneficial effects ascribed to large gut SCFAs is a decrease in intestinal digesta pH, which in turn creates an unfavourable environment for pathogenic microbiota but supports the growth of ‘good’ bacteria and nutrient absorption^39^. In presented study, the greatest reduction in SCFA concentration following the use of a high-fat, low-fibre diet was noted for caecal acetic acid, which has been proposed as a key factor in the ability of bifidobacteria to combat enteropathogens^38^. To conclude, the high-fat, low-fibre diet used in the present study created an unfavourable intestinal environment, which could adversely affect the functioning of the entire body^40^.

In the present study, the dosage 0.3 mg Cr(III)/kg BW challenged to rats was taken according to a Tolerable Daily Intake (TDI) from the Scientific Opinion on Dietary Reference Values for chromium^3^ with no observed adverse effects. In the present experiment the applied dose should be regarded as high, both in a rat diet and when calculated from animal dose to the human one. When the body surface area (BSA) normalization method is used the dose for human being is about 3 mg per day and that is 3 times higher than that found in many clinical trials with the highest Cr application. Therefore the applied in the present study Cr dose to rats should be regarded as a pharmacologically relevant one and it was chosen to the research needs of comparison commonly used Cr(III) form in supplements and novel form of chromium nanoparticles.

Intake of excessive amounts of chromium, especially for a long period, can lead to cytotoxic and genotoxic reactions disturbing the immune response^1^. In the present study, the dietary addition of chromium at the dose of 0.3 mg/kg BW, irrespective of the form used, increased the level of IL-2 in the small intestinal tissue of rats, with the most pronounced effect observed in the case of Cr-Met and Cr-NPs. Similarly, the previous research of Dworzański et al*.*^3^ showed stimulation of the immune system in the form of increased plasma levels of IL-2 in rats receiving the mentioned above pharmacologically relevant dose of Cr(III) in the form of picolinate, a methionine complex, or nanoparticles. IL-2 is produced mainly by CD4^+^ T helper (Th) cells, CD8^+^ T cells, and NK and NKT cells, and sometimes also by activated dendritic cells and mast cells^41^. It is responsible for the differentiation, proliferation and activity of lymphocytes, particularly regulatory T (Treg) cells^42^, and is also involved in promoting the apoptotic death of abnormal cells^20^. The small intestine, in which absorption processes take place, is the organ most exposed to the harmful effects of xenobiotics present in food. It can therefore be assumed that the stimulation of the immune response observed in the present study was the result of local, mild inflammation caused by direct contact between pharmacologically relevant dose of Cr present in the diet and the cells of the small intestine. This implicates the needs of further research with the lower nutritionally relevant Cr doses, especially with regard to nanoparticle form. The results of the previous research of Dworzański et al.^13^, however, in which we observed an increase in plasma IL-2 levels in rats following administration of three different forms of Cr at pharmacologically relevant dose, suggests that the addition of Cr stimulates not only a local immune response but also a systemic one. The pro-inflammatory properties of CrNPs can be explained by their small size and higher reactivity in comparison to their macro-counterparts, which significantly increases the area of their contact with the small intestinal wall and the degree of absorption into the enterocytes. On the other hand, it is very difficult to explain the analogous effect observed in the case of the Cr methionine complex, as complexes of elements with amino acids are known for their milder effects on the body than in the case of non-ionic or nanoparticle forms^43^.

The effect of Cr(III) on redox status is not yet fully understood and explained. On the one hand, chromium can have a beneficial effect on the antioxidant system, e.g. by increasing the level of reduced glutathione in the body and stimulating the activity of catalase and superoxide dismutase^44^. On the other hand, it is believed that an intracellular reduction in Cr can lead to increased free radical synthesis due to Fenton and Haber–Weiss reactions^45^. Reactive oxygen species generated in this manner can react with biomolecules in the body, leading to their oxidation and thus to the loss of specific properties and functions^46^. Damage to genetic material seems to be particularly dangerous to the body, as it can lead to mutations and the initiation of neoplastic processes^26,47^. The literature confirms the significant role of reactive oxygen species in the pathogenesis of numerous diseases of the gastrointestinal tract, including colorectal cancer^48^ and IBD^49,50^. The present study did not reveal an increase in oxidation or DNA methylation in the small intestine as a result of the addition of three different forms of chromium to the diet of rats, especially with regard to the high pharmacologically relevant dose used in the present experiment. However, significant changes were noted in the activity of DNA repair enzymes (APE-1 and TDG) as a result of chromium supplementation. These enzymes are involved in the base excision repair (BER) pathway, which is dominant in the case of oxidative DNA damage^51^. Both apurinic/apyrimidinic endonuclease 1 (APE-1) and thymine DNA glycosylase (TDG) recognize and excise damaged nitrogen bases so that they can be replaced by new, undamaged ones^52^. The literature indicates that in the case of increased ROS synthesis leading to DNA damage, the activity of repair enzymes, especially APE-1, increases^53^. This is consistent with the results of present research, in which the addition of 0.3 mg/kg BW CrNPs to the diet of rats resulted in an increase in APE-1 activity in the small intestinal tissue. It can therefore be supposed that the presence of high pharmacologically relevant dose of CrNPs in the diet increased free radical synthesis in the analysed tissue of rats. The fact that there was no increase in 8-OHdG suggests that the DNA repair mechanisms were efficient enough to remove the damaged bases without allowing their harmful oxidation products to accumulate. The study also showed that while the addition of Cr-Met to the diet of rats decreased the activity of APE-1, it increased the activity of TDG in the small intestinal wall. Ding et al.^54^ report that in the case of intensive ROS synthesis, damage to genetic material can accumulate in cells, which entails a proportional reduction in the activity of DNA repair mechanisms. This is probably due to depletion of the enzymatic activity of APE-1, which in conditions of constant mobilization is not able to regenerate sufficiently fast and effectively. In light of the above, it can be supposed that increased free radical synthesis involving Cr-Met led to an increase in the amount of DNA damage requiring the involvement of repair pathways. This caused an increase in the activity of APE-1. The reduced activity of TDG, on the other hand, may suggest that DNA repair mechanisms are slowly depleted. Considering the fact that in Cr-Met the chromium is conjugated with ligand methionine, hence the question arises whether the observed ROS formation should have been ascribed solely to the chromium dietary addition. The quite recent work by Jankowski et al.^55^ pointed at rather beneficial effects of judicious increase in dietary Met content on the antioxidant defense system in turkeys. It has been also reported that L-methionine acts as L-Cys precursor—a potent antioxidant molecule^56^.

The understanding of the interaction between the gut microbiota and host health has recently improved dramatically. However, the effects of exposure to some metals on the gut microbiota remain poorly characterized. Richardson et al.^57^ observed only modest changes to the microbiota composition of rats in response to high doses of chromium (sodium dichromate), in contrast to enormous changes following the use of arsenic, cadmium and nickel. Indeed, although all Cr forms used in the present study at pharmacologically relevant dose of 0.3 mg/kg BW efficiently reduced the extracellular activity of bacterial β-glucuronidase in the caecal digesta of rats fed a high-fat, low-fibre diet, the effect on the activity of α-glucosidase, α-galactosidase and β-galactosidase was negligible. As a result, the caecal concentration of total SCFA, including acetate and propionate, was not affected by dietary Cr. Only in the case of butyric acid were the caecal concentration and percentage in the SCFA profile noticeably reduced by the addition of Cr-Pic and Cr-NPs to the high-fat, low-fibre diet. This requires further study, as butyric acid plays a paramount role in maintaining a healthy large intestinal epithelium and colonocyte metabolism^58^. Imbalance of the intestinal microbiota has been well demonstrated in patients with obesity, non-alcoholic fatty liver disease, and type 2 diabetes^59,60^. Guo et al.^61^ showed that organic chromium derived from the chelation of *G. frondosa* polysaccharide-chromium(III) exhibited high hypoglycaemic and hypolipidaemic activity in mice with diabetes induced by a high-fat diet and streptozotocin (STZ) injection. This activity involved regulation of liver-related gene transcription and modulation of the intestinal microbiota. Previous studies on Wistar rats even showed that the gut bacteria provided the first line of defence by converting toxic Cr(VI) to the less toxic Cr(III), which may act similarly to a prebiotic^62^. The authors demonstrated that Cr(III) led to the development of large gut antibiotic resistance, with the most pronounced effects noted for *Pseudomonas* sp. and the least for *E. coli*. It would be interesting to confirm such effect for chromium(III) conjugated with organic ligands, as in the case of Cr-Pic and Cr-Met. Antibiotic resistance was also developed by *Lactobacillus* sp.^62^. Yang et al.^63^ reported positive effects of providing supplementary Cr-enriched *Bacillus subtilis* to mice, manifested by enhanced regulation of body growth, increased tissue organic Cr concentrations, beneficially altered caecal microbiota, and enhanced insulin receptor expression, leading to significant changes in plasma biochemistry. Interestingly, given the fairly minor effect of pharmacologically relevant dose of Cr on the microbial enzymatic activity in the caecum in the present study, the addition of Cr-Pic, Cr-Met, and Cr-NPs efficiently diminished the activity of the potentially harmful enzymes β-glucosidase and β-glucuronidase in the faeces collected on days 7, 14, 28 and 56 of experimental feeding. This effect was noted mainly in the case of the high-fat, low-fibre diet, with the most effective reduction in faecal β-glucosidase and β-glucuronidase activity following the use of Cr-NPs. There is of course the need to confirm or exclude similar action of chromium nanoparticles on microbial metabolic activity when the applied dose of chromium is less pharmacologically and more nutritionally relevant. The presented results dealing with the microbial response to various forms of Cr(III) support the urgent need to understand the impact of less toxic Cr(III) (vs Cr(VI)) on the microbial environment of the gastrointestinal tract^64,65^.

## Conclusion

In conclusion, the high-fat diet increased DNA oxidation and raised the level of the pro-inflammatory interleukin IL-6 in the cells of the small intestinal wall, and additionally adversely affected the morphological parameters of the small intestinal wall. The high-fat diet was shown to reduce the intensity of fermentation processes in the caecum while increasing the activity of potentially harmful enzymes in the faeces.

The addition of a pharmacologically relevant dose (0.3 mg/kg BW) of Cr in the form of Cr-NPs and Cr-Met increased mobilization of enzymes of the DNA repair pathway as the response to elevated ROS formation in the small intestinal tissue, especially in rats fed a high-fat, low-fibre diet. All forms of dietary Cr used in the study beneficially decreased the activity of caecal bacterial β-glucuronidase as well as the activity of faecal β-glucosidase and β-glucuronidase in rats fed a high-fat, low-fibre diet. This effect was especially pronounced in rats treated with Cr nanoparticles. However, due to the increase in the level of the pro-inflammatory cytokine IL-2 in the small intestinal wall of rats, induced by all tested forms of chromium, it is difficult to state conclusively that this element can mitigate unfavourable pro-inflammatory and oxidative changes induced by a high-fat diet in the small intestinal wall. However, the obtained results cannot be generalized as they may be the result of the action of not only chromium, but also additional ligands (picolinate, methionine).

## Materials and methods

The study presented in this research paper is part of a large research project to determine the effects of a high-fat low-fibre diet and various forms of chromium on many different aspects of the biological response of rats. Therefore, both the design of the experiment, the research procedures used and some physiological results have been published yet in the study of Dworzański et al.^9^, Ognik et al.^10^ and Dworzański et al.^13^.

### Forms of chromium used in the experiment

Chromium picolinate (Cr-Pic; purity > 980 g/kg) was purchased from Sigma-Aldrich Sp. z o.o. (Poznan, Poland). Chromium-methionine complex (Cr-Met) was purchased from Innobio Co., Ltd. (Siheung, Korea). Chromium nanopowder (Cr-NPs) with 99.9% purity, size 60–80 nm, spherical shape, specific surface area 6–8 m^2^/g, bulk density 0.15 g/cm^3^, and true density 8.9 g/cm^3^ was produced by and purchased from SkySpring Nanomaterials (Houston, TX, USA)^9,10,13^.

### Animals and diets

The subject of the experiment was 56 male outbred Wistar rats (*Rattus norvegicus*. Cmdb:WI) at the age of 5 weeks and with body mass of 131 ± 4 g. The rats were provided by the Institute of Animal Reproduction and Food Research PAS (No. 051 in the Registry of Laboratory Animal Breeders in Poland). The experiment was carried out in compliance with the European Guidelines for the Care and Use of Laboratory Animals^66^ and was approved by the Local Ethics Committee for Animal Experiments in Olsztyn (Approval No. 04/2019; Olsztyn. Poland). The study was carried out in compliance with the ARRIVE guidelines. Every effort was made to minimize the suffering of the animals used in the experiment.

Rats were randomly assigned to eight groups of seven animals each. They were kept for eight weeks in separate metabolic cages under a constant temperature (21–22 °C), a ventilation rate of 20 air changes per hour, and a 12/12 h light/dark cycle. They had free access to drinking water and semi-purified diets, which were modified variants of a casein diet for laboratory rodents recommended by the American Institute of Nutrition^67^. After the diets were prepared they were stored at 4 °C in hermetic containers until the experiment was completed (Table 7). Two types of diet were used. The first was a standard diet (S diets) containing 8% rapeseed oil as a fat source and 5% cellulose as a source of dietary fibre. The other (F diets) was a high-fat, low-fibre modification of the S diets, with 17% lard replacing corn starch and only 3% cellulose content. All diets had equal content of dietary protein from a casein preparation (20% of diet; Lacpol Co., Murowana Goslina, Poland) and DL-methionine (0.3% of diet), but the caloric density of the F diets was 23% higher than that of the S diets due to their increased fat content (25% vs. 8%). The mean measured Cr content in the diets without supplementation was 1.24 mg/kg, while in the diets with Cr-Pic, Cr-Met and Cr-NP—3.99, 4.02 and 4.03 mg/kg, respectively^68^. The chromium sources were added in the same amount to the S and F diets, resulting in a two-factorial design (see ‘Statistical analysis’ below). Each rat received chromium in the amount of 0.3 mg/kg BW, a dosage selected according to the recommendation of the EFSA NDA Panel^3^ and this is the pharmacologically relevant dose of additional Cr in the diet. The following sources of Cr were used in the diets: chromium(III) picolinate (Cr-Pic), chromium(III)-methionine (Cr-Met), and chromium nanoparticles (Cr-NPs). To ensure the safety of the individual preparing the diets containing Cr nanoparticles, while maintaining comparable conditions for all treatments, all Cr sources were added in the form of an emulsion with rapeseed oil instead of in the mineral mixture.

**Table 7:** Composition of diets fed to rats (%).

Ingredient/group	S	F
Casein^1^	20.0	20.0
DL-methionine	0.3	0.3
Celullose^2^	5.0	3.00
Sucrose	10.0	10.0
Rapeseed oil	8.0	8.0
Lard	–	17.0
Vitamin mixture^3^	1.0	1.0
Mineral mixture^4^	3.5	3.5
Choline chloride	0.2	0.2
Cholesterol	0.3	0.3
Corn starch^6^	51.7	36.7
**Calculated nutritional value**		
Protein, kcal%	20.3	12.0
Fat, kcal%	18.0	33.2
Carbohydrates, kcal%	61.7	54.8
Energy density, kcal/gm	4.00	6.78

^1^Casein preparation (LACPOL Co., Murowana Goslina, Poland), crude protein 89.7%, crude fat 0.3%, ash 2.0% water 8.0%.

^2^α-cellulose (SIGMA. Poznan. Poland)—main source of dietary fibre.

^3^AIN-93G-VM^28^; g/kg mix: 3.0 nicotinic acid, 1.6 Ca pantothenate, 0.7 pyridoxine–HCl, 0.6 thiamine-HCl, 0.6 riboflavin, 0.2 folic acid, 0.02 biotin, 2.5 vitamin B_12_ (cyanocobalamin, 0.1% in mannitol), 15.0 vitamin E (all-rac-α-tocopheryl acetate, 500 IU/g), 0.8 vitamin A (all-trans-retinyl palmitate, 500 000 IU/g), 0.25 vitamin D_3_ (cholecalciferol, 400 000 IU/g), 0.075 vitamin K_1_ (phylloquinone), 974.655 powdered sucrose.

^4^Mineral mix; g/kg mix: 357 calcium carbonate anhydrous CaCO_3_, 196 dipotassium phosphate K_2_HPO_4_, 70.78 potassium citrate C_6_H_5_K_3_O_7_, 74 sodium chloride NaCl, 46.6 potassium sulphate K_2_SO_4_, 24 magnesium oxide MgO, 18 microelement mixture^5^, starch to 1 kg = 213.62.

^5^Microelement mixture; g/kg mix: 31 iron (III) citrate (16.7% Fe), 4.5 zinc carbonate ZnCO_3_ (56% Zn), 23.4 manganese (II) carbonate MnCO_3_ (44.4% Mn), copper carbonate CuCO_3_ (55.5% Cu), 0.04 potassium iodide KI, citric acid C_6_H_8_O_7_ to 100 g.

^6^Corn starch preparation: crude protein 0.6%, crude fat 0.9%, ash 0.2%, total dietary fibre 0%, water 8.8%.

Experimental sources of dietary Cr: chromium(III) picolinate (Cr-Pic), chromium(III)-methionine (Cr-Met), nano-sized chromium (Cr-NPs) were added to the an emulsion together with dietary rapeseed oil, not in the mineral mixture (MX).

S—Standard diet; F—high-fat, low-fibre diet.

### Sample collection and analyses

At the termination of the experiment, the rats were anaesthetized intraperitoneally with ketamine/xylazine solution in 0.9% NaCl (100/10 mg per kg BW) according to recommendations for euthanasia of experimental animals. Following laparotomy, the small intestine and caecum were removed and weighed. The small intestine was divided into four equal parts. The fourth part (ileum) from the stomach side was rinsed with ice-cold physiological saline and frozen in liquid nitrogen. Samples of caecal contents were used for immediate analysis of short-chain fatty acids (SCFAs), while the remainder of the caecal digesta was transferred to tubes and stored at – 70 °C.

### Ex vivo analysis

#### Analysis of immune status in ileal tissue

Levels of globulin, immunoglobulins A and G (IgA and IgG), interleukin-6 (IL-6), interleukin-2 (IL-2), and tumour necrosis factor α (TNF-α), as well as the activity of ceruloplasmin (Cp), were determined in the ileal tissue. Immune parameters were determined using commercial enzyme-linked immunosorbent assay (ELISA) kits (MyBioSource Inc., San Diego, USA). Absorbances were measured at 450 nm with an ELISA reader.

#### Analysis of oxidative and epigenetic damage to DNA and activity of repair enzymes in ileal tissues

DNA was isolated from the intestinal wall using QIAGEN kits. Epigenetic changes in the intestinal wall were determined by analysing global DNA methylation (methylome) using Sigma Aldrich diagnostic kits. The levels of 8-hydroxydeoxyguanosine (8-OHdG) and the activity of apurinic/apyrimidinic endonuclease 1 (APE-1), thymine DNA glycosylase (TDG), and DNA-3-methyladenine glycosylase (ANPG) were determined in the ileal wall using OxiSelect diagnostic kits (Cell Biolabs, Inc., San Diego, USA).

#### Analysis of fermentation processes in the caecum

Caecal digesta samples were subjected to SCFA analysis using gas chromatography (Shimadzu GC-2010; Shimadzu, Kyoto, Japan). The samples (0.2 g) were mixed with 0.2 mL formic acid, diluted with deionized water, and centrifuged at 7211×*g* for 10 min. The supernatant was loaded onto a capillary column (SGE BP21, 30 m × 0.53 mm) using an on-column injector. The initial oven temperature was 85 °C, raised to 180 °C by 8 °C/min, and held there for 3 min. The temperatures of the flame ionization detector and the injection port were 180 °C and 85 °C, respectively. The sample volume for GC analysis was 1 µL. The activity of microbial enzymes (α- and β-glucosidase, α- and β-galactosidase, and β-glucuronidase) released from bacterial cells into the faecal/caecal environment was analysed by the rate of *p*- or *o*-nitrophenol release from respective nitrophenylglucosides, as described previously^69^. The faecal samples were collected at 0, 7, 14, 28, 56 day of feeding while the caecal ones at the termination of the study. The enzyme activity was expressed as μmol of product formed per hour per g of digesta/faeces.

### Statistical analysis

The results are presented in tables as means and pooled SEM. A two-way ANOVA was used to determine the effect of the two main factors, i.e. the Cr source (Cr: none, Cr-Pic, Cr-Met, or Cr-NPs) and the diet type (D: standard or high-fat, low-fibre diets), and the interaction effect between them (Cr × D). In the case of a significant interaction (*P* ≤ 0.05), the differences between all groups were then determined using Duncan’s post hoc test at *P* ≤ 0.05. The data were checked for normality prior to the statistical analyses. The statistical analysis was performed using STATISTICA software version 10.0 (StatSoft Corp. Krakow, Poland)^9,10,13^.
